# Deciphering genetic diversity and inheritance of tomato fruit weight and composition through a systems biology approach

**DOI:** 10.1093/jxb/ert349

**Published:** 2013-10-22

**Authors:** Laura Pascual, Jiaxin Xu, Benoît Biais, Mickaël Maucourt, Patricia Ballias, Stéphane Bernillon, Catherine Deborde, Daniel Jacob, Aurore Desgroux, Mireille Faurobert, Jean-Paul Bouchet, Yves Gibon, Annick Moing, Mathilde Causse

**Affiliations:** ^1^INRA, UR1052, Unité de Génétique et Amélioration des Fruits et Légumes, F-84143 Avignon, France; ^2^Northwest A&F University, College of Horticulture, Yang Ling, Shaanxin 712100, PR China; ^3^INRA-UMR 1332 Biologie du Fruit et Pathologie, Centre INRA de Bordeaux, F-33140 Villenave d’Ornon, France; ^4^Université de Bordeaux, UMR1332 Biologie du Fruit et Pathologie, Centre INRA de Bordeaux, F-33140 Villenave d’Ornon, France; ^5^Metabolome Facility of Bordeaux Functional Genomics Center, IBVM, Centre INRA de Bordeaux, F-33140 Villenave d’Ornon, France

**Keywords:** Fruit, metabolome, proteome, systems biology, tomato.

## Abstract

Integrative systems biology proposes new approaches to decipher the variation of phenotypic traits. In an effort to link the genetic variation and the physiological and molecular bases of fruit composition, the proteome (424 protein spots), metabolome (26 compounds), enzymatic profile (26 enzymes), and phenotypes of eight tomato accessions, covering the genetic diversity of the species, and four of their F_1_ hybrids, were characterized at two fruit developmental stages (cell expansion and orange-red). The contents of metabolites varied among the genetic backgrounds, while enzyme profiles were less variable, particularly at the cell expansion stage. Frequent genotype by stage interactions suggested that the trends observed for one accession at a physiological level may change in another accession. In agreement with this, the inheritance modes varied between crosses and stages. Although additivity was predominant, 40% of the traits were non-additively inherited. Relationships among traits revealed associations between different levels of expression and provided information on several key proteins. Notably, the role of frucktokinase, invertase, and cysteine synthase in the variation of metabolites was highlighted. Several stress-related proteins also appeared related to fruit weight differences. These key proteins might be targets for improving metabolite contents of the fruit. This systems biology approach provides better understanding of networks controlling the genetic variation of tomato fruit composition. In addition, the wide data sets generated provide an ideal framework to develop innovative integrated hypothesis and will be highly valuable for the research community.

## Introduction

Identifying the genes controlling the variation of complex traits is a key goal of evolutionary genetics and plant biology. Attempts to identify genetic variants underlying quantitative traits have been achieved by traditional linkage mapping and genome-wide association studies using molecular markers. However, resolution of quantitative trait loci (QTLs) is limited and the identification of the polymorphisms responsible for the variation is not straightforward. Furthermore, as several intermediate levels interact between the genotypes and the phenotypes, DNA sequence variation [single nucleotide polymorphisms (SNPs) or Indels] may not directly affect the traits. Intermediate molecular phenotypes such as gene expression, protein abundance, and metabolite concentration also vary in populations and are themselves quantitatively inherited (Rockman and Kruglyak, 2006). Nowadays, rapid technological advances in high-density experiments such as next-generation sequencing (NGS), RNA expression analysis through microarray or RNAseq, mass spectrometry (MS) coupled to gas chromatography (GC-MS) or to liquid chromatography (LC-MS), and nuclear magnetic resonance (NMR) metabolic profiling enable scientists to obtain large exhaustive data sets and analyse biological systems as a whole. Integration of the genome expression products at different levels should help in dissecting the genetic variation of a given quantitative trait.

Systems biology relates the variation analysed at different expression levels, from phenotype to metabolome and proteome, studying the behaviour of all the elements in a biological system (Gutierez *et al.*, 2008; Saito and Matsuda, 2010). A bottom-up systems biology approach consists of integrating ‘omic’ resources (genomic, transcriptomic, proteomic, and metabolomic) and large physiological data sets, together with statistical network analysis in order to identify candidate genes underlying phenotypes and to construct complex regulation networks (Kliebenstein, 2010). This approach was first applied to yeast by combining DNA microarrays and quantitative proteomics to describe the galactose pathway (Ideker *et al.*, 2001). It was then applied to gene expression analysis in *Escherichia coli* (Rosenfeld *et al.*, 2002), and to *Arabidopsis* by Hirai *et al.* (2005) who elucidated gene to gene and metabolite to gene networks by integrating metabolomic and transcriptomic data. Systems biology has also been used to study the natural genetic variation at different levels, such as metabolomics (Keurentjes, 2009; Kliebenstein, 2009*a*), proteomics (Stylianou *et al.*, 2008), and transcriptomics (Keurentjes *et al.*, 2008; Kliebenstein, 2009*b*).

Tomato (*Solanum lycopersicum*) is the model species for the study of fleshy fruit development and composition (Giovannoni *et al.*, 2004). It is a self-pollinated species and is derived from its closest wild ancestor *Solanum pimpinellifolium* (Nesbitt and Tanksley, 2002). Cherry tomato accessions (*Solanum lycopersicum* var. *cerasiforme*) have an intermediate position between these two species, as their genome is a mosaic of those from *S. lycopersicum* and *S. pimpinellifolium* (Ranc *et al.*, 2008). During tomato domestication, the diversification of fruit aspect, as well as the adaptation to a wide range of environmental conditions was simultaneous with a strong reduction of molecular diversity (Miller and Tanksley, 1990; Blanca *et al.*, 2012). This lack of genetic variation in cultivated species led geneticists to study trait variation mostly in distant crosses involving wild species, and thus limited the exploitation of intraspecific variation. Today the availability of the tomato genome sequence (Tomato Genome Consortium, 2012) and of a large number of SNP markers (Sim *et al.*, 2012) allows a re-examination of the variation and inheritance of agronomical and fruit traits at the intraspecific level.

Systems biology approaches have been used in tomato to study fruit development. Carrari *et al.* (2006) and Mounet *et al.* (2009) analysed transcriptome and metabolome variation during fruit development. Garcia *et al.* (2009) combined phenotype, metabolome, transcriptome, and proteome profiles to study genes related to ascorbate metabolism in three transgenic lines. Wang *et al.* (2009) compared the transcriptome and metabolome to uncover the molecular events underlying fruit set, while Osorio *et al.* (2011) compared enzyme activity, metabolite and transcript profiles to analyse the connectivity between these groups of traits in fruit ripening mutants. However, these studies were only focused on a few mutants or on the effect of introgression in *S. lycopersicum* of wild species alleles. Little is known about the genetic variation in metabolic, enzymatic, and proteomic profiles contributing to phenotypic trait variation in the species.

In the present study, the aim was to decipher the complex relationships between several successive levels of omic profiles to characterize the genetic variation and physiological bases of quantitative traits in tomato fruit. For this purpose, the variation of eight genotypes representing a large range of phenotypic and genotypic diversity (four *S. lycopersicum* and four *S. lycopersicum* var. *cerasiforme*) and four of their corresponding hybrids was compared at two stages of fruit development [cell expansion (CE) and orange-red (OR)]. Their metabolic, enzymatic, and proteome profiles were characterized. Genetic variability was analysed for all traits, and inheritance patterns of traits that were significantly different among genotypes were assessed. Relationships among traits were analysed within and between each group of traits at each stage, and networks were constructed using sparse partial least square (sPLS) regression. This systems biology approach combining proteome, metabolome, and phenotypic analysis gave insights into the diversity and relationships of quantitative traits at different levels.

## Materials and methods

### Plant materials

Eight tomato lines comprising four *S. lycopersicum* accessions (Levovil, Stupicke Polni Rane, LA0147, and Ferum) and four *S. lycopersicum lycopersicum* var. *cerasiforme* accessions (Cervil, Criollo, Plovdiv24A, and LA1420), and four of their corresponding F_1_ hybrids (Levovil×Cevil, Stupicke Polni Rane×Criollo, LA0147×Plovdiv24A, and Ferum×LA1420) were used in this study (details of the accessions are shown in Supplementary Table S1 and Supplementary Fig. S1 available at *JXB* online). Lines were selected, based on a previous molecular characterization of 360 tomato accessions (Ranc *et al.*, 2008), to include the maximum genetic diversity of the species. Xu *et al.* (2013*b*) genotyped these lines and the line sequenced to obtain the reference genome (Heinz1706, Tomato Genome Consortium, 2012). From the 139 SNP markers characterized, 133 were polymorphic (96%), showing the large range of molecular diversity represented by the eight lines. The range of polymorphism between the lines and the reference genome (Heinz1706) ranged from 27% to 82% (Supplementary Table S2) The genetic distances among the parents of F_1_ hybrids were variable. According to data of Xu *et al.* (2013*b*), Levovil and Cervil were the two most distant accessions (82% SNP polymorphic), followed by LA0147×Plovdiv 24A (40%), Stupicke Polni Rane×Criollo (34%), and Ferum×LA1420 (27%).

Plants were grown during spring 2010 under greenhouse conditions (16/20 °C) in Avignon (South of France). Plants were separated into two blocks; five plants per genotype were included in each block.

For proteome, metabolome, and enzymatic measurement, two stages of development, CE and OR, were selected. The CE stage was chosen as a representative stage of the growing tomato fruit; the OR stage was chosen because it is unequivocally determined and is the key step where enzyme and protein concentrations are changing and will determine the final characteristics of the fruit. The number of days after anthesis to reach cell expansion varied among genotypes depending on their fruit size. Thus CE sampling was done at 14, 20, or 25 d after anthesis for small (Cervil), medium (Criollo, Plovdiv 24A, Stupicke Polni Rane, and the four F_1_ hybrids), or large (LA0147, Levovil, and Ferum) fruited accessions, respectively. OR sampling was done based on fruit colour change. Three biological replicates by stage were analysed. Each replicate included 7–20 fruits from both greenhouse blocks to buffer environmental variations. Fruit pericarps were collected, immediately frozen, ground in liquid nitrogen, and stored at –80 °C until analysis. For fruit phenotypic trait measurements, five fruits were harvested from the 10 plants of each genotype at the following six stages: (i) CE stage; (ii) CE+7 d; (iii) CE+14 d; (iv) CE+21 d; (v) OR stage; and (vi) red ripe. Fruits were evaluated for fresh weight (FW), fruit diameter (FD; measured using a caliper), and dry matter content (DMC). DMC (expressed in g 100g^–1^ FW) was assessed after 5 d in a ventilated oven at 80 °C.

### Metabolome and enzyme activity analysis

Metabolome analyses were performed at the Metabolome Facility of Bordeaux, using quantitative proton NMR (^1^H-NMR) profiling of polar extracts and liquid chromatography quadrupole time-of-flight tandem mass spectrometry (LC-QTOF-MS) profiling of semi-polar extracts. ^1^H-NMR profiling was performed as described in Deborde *et al.* (2009) with minor modifications. Briefly, polar metabolites were extracted on lyophilized powder (50mg DW per biological replicate) with an ethanol–water series at 80 °C. The lyophilized extracts were titrated to pH 6 and lyophilized again. Each dried titrated extract was solubilized in 0.5ml of D_2_O with (trimethylsilyl) propionic-2,2,3,3-d_4_ acid (TSP) sodium salt (0.01% final concentration) for chemical shift calibration and EDTA (5mM final concentration for the CE and 2mM for the OR stage). ^1^H-NMR spectra were recorded at 500.162 MHz on a Bruker Avance III spectrometer (Bruker, Karlsruhe, Germany) using an ATMA inverse 5mm probe flushed with nitrogen gas and an electronic reference for quantification (ERETIC2). Sixty-four scans of 32 000 data points each were acquired with a 90 ° pulse angle, a 6000 Hz spectral width, a 2.73 s acquisition time, and a 25 s recycle delay. Two technological replicates were used per biological replicate. Preliminary data processing was conducted with TOPSPIN 3.0 software (Bruker Biospin, Wissembourg, France). The assignments of metabolites in the ^1^H-NMR spectra were made by comparing the proton chemical shifts with values of the MeRy-B metabolomic database (Ferry-Dumazet *et al.*, 2011), by comparison with spectra of authentic compounds recorded under the same solvent conditions, and/or by spiking the samples. The metabolite concentrations were calculated using AMIX (version 3.9.7, Bruker) software. The 144 ^1^H-NMR spectra of the data set were converted into JCAMP-DX format and deposited with associated metadata into the Metabolomics Repository of Bordeaux MeRy-B (Ferry-Dumazet *et al.*, 2011, http://www.cbib.u-bordeaux2.fr/MERYB/view/project/34, accessed September 2013).

LC-QTOF-MS profiling of aqueous methanol–0.1% formic acid extracts was performed from lyophilized powder (20mg in 1ml). For each biological replicate, two extractions were performed and two injections per extract were used. An Ultimate 3000 HPLC (Dionex, Sunnyvale, CA, USA) was used to separate metabolites on a reversed phase C18 column (150×2.0mm, 3 µm; Phenomenex, Torrance, CA, USA) using a 30min linear gradient from 3% to 95% acetonitrile in water acidified with 0.1% formic acid. Metabolites were detected using a QTOF mass spectrometer (Bruker). Electrospray ionization in positive mode was used to ionize the compounds. Scan rate for ions at *m/z* range 100–1500 was fixed at two spectra per second. Methyl vanillate was spiked in the extraction solvent and used as an internal standard. One sample was used as a quality control sample and injected after every 10 injections. Raw data were processed in a targeted manner using QuantAnalysis 2.0 software (Bruker). This resulted in eight compounds identified based on accurate mass measurement and comparison with data from Gomez-Romero *et al.* (2010).

Ascorbic acid was measured using a spectrofluorometric method and values were expressed as total ascorbate (ascorbic acid+dehydroascorbate) as previously described by Stevens *et al.* (2007). The maximum activity (*V*_max_) of 26 enzymes of primary metabolism was assayed using a robotized platform as described in Gibon *et al.* (2004) and in Steinhauser *et al.* (2010). Supplementary Tables S3 and S4 at *JXB* online present the lists of the primary and secondary metabolites and the enzyme activities analysed.

### Proteome analysis

Methods for protein extraction, two-dimensional gel electrophoresis (2-DE), and protein identification and classification were as detailed in Xu *et al.* (2013*a*). Briefly, proteins were extracted using the phenol extraction method developed by Faurobert *et al.* (2007). Later, proteins were separated by 2-DE. After Coomassie colloidal staining, image analysis was performed with Samespot software (version 4.1), and the normalized spot volumes were obtained. Protein identification of 424 variable spots was performed at the proteome platform of Le Moulon (Gif-sur-Yvette) using a nano-LC-MS/MS method following the procedure described in Xu *et al.* (2013*a*). The database search was run against the International Tomato Annotation Group (ITAG) Release 2.3 of predicted proteins (SL2.40) database (http://solgenomics.net/, accessed September 2013) with X!Tandem software (http://www.thegpm.org/TANDEM/, version 2010.12.01.1). The Fasta sequence of the identified proteins was employed to re-annotate the proteins using the Blast2GO package (Conesa *et al.*, 2005). Sequences were compared against the NCBI-nr (version April 9, 2012) database of non-redundant protein sequence using BLASTX with the default settings.

### Statistical analysis and inheritance analysis

Metabolite contents and enzyme activities were expressed on a dry weight basis to be comparable. All the analyses were performed using R (R Development Core Team, 2012, http://www.R-project.org/, accessed September 2013).

Data were submitted to a two-way analysis of variance (ANOVA; *P* < 0.05) with genotype, stage, and interaction effect, and then to one-way ANOVA with genotype effect at each stage. In addition, to assess the mode of inheritance of the traits, one-way ANOVA was also performed with genotype effect for each cross (two parental lines and their hybrid) and stage.

Means and standard deviations were calculated for each trait (phenotypic traits, metabolite contents, enzyme activities, and protein spot volumes) in each genotype and stage. Significantly different (*P* < 0.05) traits in each cross were selected at each stage to estimate additive (A) and dominance (D) components of genetic variation. A is equivalent to half of the difference between two parental lines. The *S. lycopersicum* line was systematically the first parent in a cross. D is the difference between the hybrid value and the parental mean. The inheritance pattern of each trait was then assessed by the dominance/additivity (D/A) ratio and classified as over-recessive (OR; D/A < –1.2), recessive (R; –1.2 ≤ D/A ≤ –0.8), additive (A; –0.8 < D/A < 0.8), dominant (D; 0.8 ≤ D/A ≤ 1.2), or overdominant (OD; D/A >1.2).

Means of metabolite contents, enzyme activities, and protein spot volumes were centred and scaled to variance unit and used for the rest of the analysis. Principal component analyses (PCAs) were performed for metabolites, enzymes, and phenotypic traits, as well as for protein spot volumes for both development stages and at each stage, with the ‘pcaMethods’ package (Stacklies *et al.*, 2007). Pearson correlations and *P*-values were calculated between significantly variable traits at each stage. Correlations were considered to be significant when |*r*| >0.7 (*P*-value <0.01). Significant correlations were plotted using the R ‘corrplot’ package (Wei, 2012, http://CRAN.R-project.org/package=corrplot, accessed September 2013). To analyse the relationships among protein spot volumes and other traits, networks were reconstructed and visualized using sPLS correlation regression analysis with the ‘mixOmics’ package (Lé Cao *et al.*, 2009). An arbitrary threshold of 0.7 was employed for network reconstruction. Nodes represent the different traits, and edges represent the relationships between variables belonging to different levels.

## Results

**Fig. 1.**
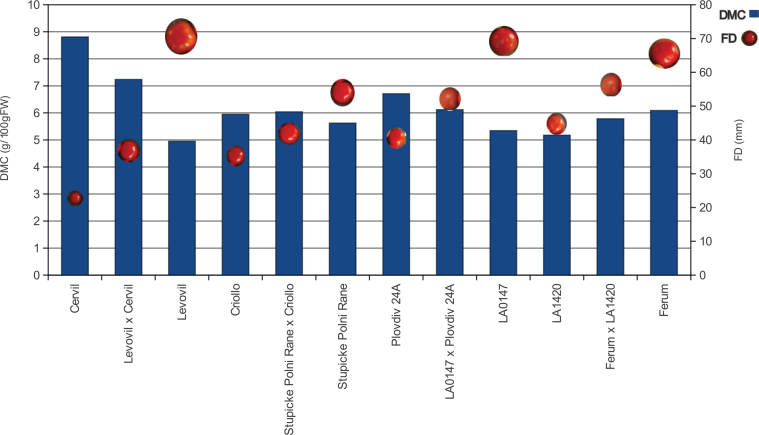
Fruit size (FD) and dry matter content (DMC) of the eight tomato accessions studied and their four hybrids (measured at the orange-red stage). (This figure is available in colour at *JXB* online.)

To represent a large range of the genetic diversity, eight tomato accessions were chosen according to previous studies (Ranc *et al.*, 2008; Xu *et al.*, 2013*b*). The eight accessions and their four hybrids were characterized at phenotypic, metabolic, and proteomic levels. The final fruit weight of the eight parental lines and the four hybrids ranged from 5.3g to 134.4g. Fruit weights of the four hybrids were intermediate between the values of their parental lines throughout fruit development (Supplementary Fig. S2 at *JXB* online). Fruit diameter (Fig. 1) was highly correlated to fruit weight, as fruits were round. DMC also showed a wide range of variation (Fig. 1; Supplementary Table S5).

### Metabolome, enzyme, and proteome profiles strongly differ among accessions

**Fig. 2.**
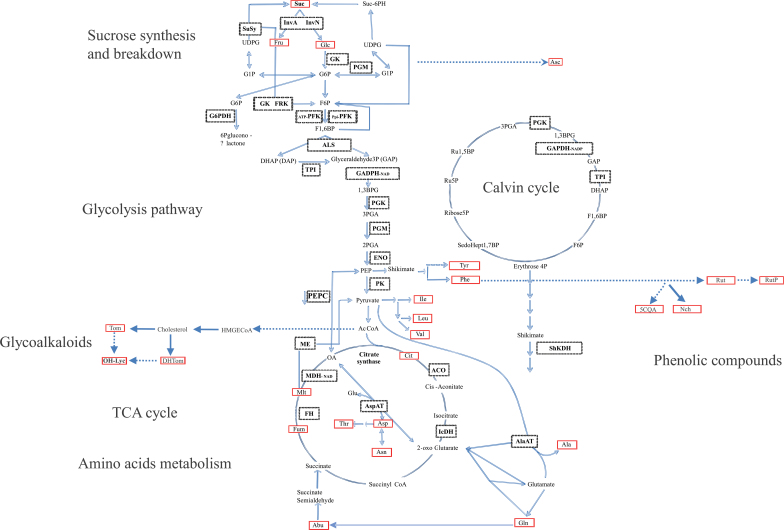
Assignment of the metabolites and enzymes studied to pathways. A total of 26 metabolites are indicated in boxes with solid lines, and 26 enzymes are highlighted in boxes with dotted lines. (This figure is available in colour at *JXB* online.)

Metabolome profiling by ^1^H-NMR and LC-QTOF-MS allowed the quantification of 18 metabolites from central carbon metabolism and eight secondary metabolites (Supplementary Table S3 at *JXB* online). In addition, 26 enzyme activities were assessed by robotized assays (Supplementary Table S4). These analyses provided a detailed characterization of sugar, organic acid, and amino acid metabolism pathways, as well as those of glycoalkaloids and phenolic compounds (Fig. 2). Proteins were isolated following 2-DE. A total of 1230 protein spots were detected. A subset of 424 spots whose abundance was significantly different between genotypes or stages were sequenced by LC-MS/MS. A total of 422 spots were identified (Xu *et al.*, 2013*a*). Supplementary Table S5 lists the mean and standard deviation of every trait for each genotype and stage.

The 12 accessions differed for most of the metabolites and phenotypic traits according to the ANOVAs (Table 1). The means of most of the traits (27/29) were significantly different across stages. The content of glucose, fructose, citrate, asparagine, aspartate,and phenylalanine increased from CE to OR, while the other amino acids decreased. The interactions between stage and genotype were significant for 93% of 29 metabolite and phenotypic traits, and 50% of these traits showed a different trend according to the genotype. The data were thus analysed stage by stage (Table 1). A large range of variability was observed among the 12 genotypes at each stage as all the trait means were significantly different, except for that of crypto-chlorogenic acid. The fold change difference between genotypes reached values as high as 5.6 for threonine content at CE or 7.9 for malate at OR.

**Table 1.** Analysis of variation for phenotypic and metabolite contents in eight tomato accessions and four F_1_ hybrids

Traits	Global analysis			CE/OR		CE stage				OR stage			
	Fs	Fg	Fgxs	Min	Max	Fg	Min	Max	Max/min	Fg	Min	Max	Max/min
FW	***	***	***	0.10	0.41	***	1.26	30.84	24.41	***	5.33	134.36	25.22
FD	***	***	***	0.46	0.74	***	14.19	42.50	3.00	***	22.73	69.95	3.08
DMC	***	***	NS	1.12	1.42	***	6.16	10.59	1.72	***	4.95	8.81	1.78
Glc	***	***	***	0.42	0.90	***	68 996.30	193 536.82	2.81	***	158574.12	233 208.35	1.47
Suc	***	***	***	0.31	1.08	***	6417.25	11 856.59	1.85	***	7596.82	28 049.81	3.69
Fru	***	***	***	0.38	0.85	***	65 853.14	198 775.33	3.02	***	173280.21	238 532.61	1.38
Ala	***	***	***	0.79	5.51	***	288.06	1356.30	4.71	***	171.01	492.55	2.88
Asn	***	***	***	0.31	0.95	***	752.84	2461.92	3.27	***	1200.65	4593.94	3.83
Asp	***	***	***	0.20	0.41	***	579.58	1278.39	2.21	***	1564.39	3598.28	2.30
Abu	***	***	***	0.80	2.25	***	4099.47	8485.63	2.07	***	2130.82	5854.16	2.75
Gln	***	***	***	0.48	1.45	***	5976.22	26 264.35	4.39	***	6605.55	24 155.80	3.66
Ile	**	***	***	0.57	3.30	***	168.70	865.48	5.13	***	232.59	687.61	2.96
Leu	***	***	***	0.40	1.26	***	306.62	927.64	3.03	***	401.47	1042.48	2.60
Phe	***	***	***	0.35	0.94	***	1209.44	4905.71	4.06	***	1988.74	7429.73	3.74
Tyr	***	***	***	0.50	2.20	***	153.13	760.58	4.97	***	185.86	656.84	3.53
Val	***	***	***	1.15	10.95	***	206.82	1004.13	4.86	***	91.66	396.69	4.33
Thr	***	***	***	0.38	2.12	***	125.21	745.30	5.95	***	221.38	768.59	3.47
Asc	***	***	***	0.48	1.13	***	1304.64	2236.94	1.71	***	1509.86	3021.01	2.00
Cit	***	***	***	0.37	0.57	***	26 295.26	62 463.29	2.38	***	49 525.87	149 942.47	3.03
Mlt	NS	***	***	0.59	3.78	***	13 562.91	25 447.13	1.88	***	3894.13	30 910.20	7.94
Fum	***	***	***	0.88	NA	***	5.54	18.71	3.38	***	0.00	12.75	NA
Tom*	***	***	***	22.16	154.19	***	983 224.09	4 556 008.75	4.63	***	16 992.93	98 910.75	5.82
DHTom*	***	***	***	9.15	63.02	***	196 588.35	2 644 852.27	13.45	***	7239.96	80 840.49	11.17
OH Lyc*	***	***	***	0.19	1.03	***	4384.91	9953.68	2.27	***	5766.39	46972.34	8.15
5CQA*	***	***	***	0.91	1.62	***	51 444.47	111 458.40	2.17	**	43 965.78	68 607.80	1.56
3CQA*	NS	NS	NS	0.45	2.33	NS	4404.24	19 467.52	4.42	NS	5977.49	13 005.07	2.18
Nch*	***	***	***	0.00	0.30	*	408.94	2137.62	5.23	***	2788.25	684 629.20	245.54
Rut*	***	***	***	0.63	4.26	***	63 505.59	243 368.28	3.83	***	28 927.20	265 669.06	9.18
RutP*	***	***	***	0.40	1.09	***	21497.91	59 076.96	2.75	***	25 248.73	79 202.85	3.14

See Supplementary Table S2 at JXB online for metabolite abbreviations.

Fs, significance level of the ANOVA for the stage factor; Fg, significance level of the ANOVA for genotype factor; Fgxs, significance level of the ANOVA for the interaction between genotype and stage; Min, minimum average values for each variable among the 12 genotypes; Max, maximum average values for each variable among the 12 genotypes; CE/OR, ratio of cell expansion and orange-red stages (min and max) for each genotype; NA, not available; NS, non-significant (*P* > 0.05).

*0.01 <*P* <0.05; **0.001 <*P* <0.01; ***P < 0.001.

The activity of 26 enzymes from central carbon metabolism, including enzymes of the Calvin cycle, glycolysis, sucrose metabolism, the tricarboxylic acid (TCA) cycle, and amino acid metabolism, was quantified and expressed relative to dry weight to be comparable with the metabolome and proteome. Enzyme activities exhibited a lower range of variation than metabolites (Table 2). The greatest differences were found between stages, where all the enzyme activities difered except that of alanine aminotransferase, fumarase, and glyceraldehyde-3-phosphate dehydrogenase (NADP). The activity of 15 enzymes was greater at CE. Two and 13 enzyme activities were significantly different among accessions at CE and OR, respectively.

**Table 2.** Analysis of variation for enzyme activities in eight tomato accessions and four F_1_ hybrids

Enzymes	Global analysis			CE/OR		Cell expansion stage				Orange-red stage			
	Fs	Fg	Fgxs	Min	Max	Fg	Min	Max	Max/min	Fg	Min	Max	Max/min
PEPC	***	NS	NS	1.63	5.01	NS	4743.07	10 166.94	2.14	NS	1393.72	3988.74	2.86
ALS	***	*	NS	1.11	6.96	NS	23 342.95	39 076.09	1.67	*	4955.98	27 286.39	5.51
G6PDH	***	NS	NS	1.41	3.22	NS	1948.75	2991.71	1.54	**	736.48	1494.57	2.03
PGM	***	NS	NS	1.56	3.58	NS	18 767.93	31 388.58	1.67	NS	6272.80	14 070.21	2.24
PK	***	NS	NS	0.98	3.04	NS	4214.45	7365.46	1.75	*	1736.57	4838.01	2.79
Ppi-PFK	***	NS	NS	1.42	5.97	NS	7380.06	13 238.75	1.79	***	1778.74	6372.06	3.58
ACO	***	NS	NS	0.85	11.98	NS	1115.56	3838.66	3.44	**	125.70	1940.54	15.44
ATP-PFK	***	NS	NS	0.71	2.05	NS	922.01	1640.44	1.78	NS	709.66	1295.85	1.83
FRK	***	*	**	1.29	8.99	*	1027.06	5020.16	4.89	NS	309.56	1373.80	4.44
InvN	***	***	**	0.12	1.60	NS	881.42	4517.10	5.12	***	1798.65	13 163.36	7.32
InvA	***	NS	NS	0.12	1.03	NS	1769.30	5805.68	3.28	*	3542.76	22 718.43	6.41
NAD-MDH	***	NS	NS	1.57	4.69	NS	212 499.39	372 186.20	1.75	**	65 345.21	186 895.32	2.86
AlaAT	NS	NS	NS	0.30	2.83	NS	8355.30	27841.06	3.33	NS	3908.25	60 730.09	15.54
FH	NS	NS	NS	0.59	10.56	NS	2227.13	7340.03	3.30	NS	266.42	5426.77	20.37
AspAT	***	NS	NS	0.63	2.75	NS	31619.55	88 013.71	2.78	NS	23 952.50	55 452.74	2.32
NAD-ME	**	NS	NS	0.89	3.06	NS	4699.19	10 772.77	2.29	NS	3248.43	9464.11	2.91
NADP-ME	***	***	**	0.59	4.00	**	1585.95	5869.58	3.70	**	960.60	3947.79	4.11
GAPDH (NAD)	***	NS	NS	1.33	5.27	NS	22 716.25	37 476.27	1.65	NS	7059.38	24 964.31	3.54
GAPDH (NADP)	NS	NS	NS	0.43	7.19	NS	4232.25	11 154.22	2.64	*	966.42	22 461.89	23.24
GK	***	NS	NS	2.57	7.59	NS	979.49	1914.96	1.96	NS	166.27	443.60	2.67
IcDH	***	**	***	0.39	1.54	NS	1210.69	31 18.82	2.58	***	1482.13	4029.86	2.72
ENO	***	NS	*	1.79	7.08	NS	3420.96	66 43.23	1.94	**	773.15	2156.06	2.79
TPI	***	NS	NS	1.64	6.26	NS	627 079.82	1 212 604.25	1.93	NS	187 195.55	505 691.43	2.70
PGK	***	NS	NS	0.72	7.58	NS	52 497.52	105 333.98	2.01	NS	13 903.37	73 080.01	5.26
SuSy	**	NS	NS	0.66	4.78	NS	1857.82	11 726.22	6.31	NS	1520.16	7106.26	4.67
ShKDH	***	**	NS	1.07	2.57	NS	1568.76	2787.32	1.78	***	725.68	2212.66	3.05

See Supplementary Table S3 at *JXB* online for enzyme abbreviations.

Fs, significance level of the ANOVA for the stage factor; Fg, significance level of the ANOVA for genotype factor; Fgxs, significance level of the ANOVA for the interaction between genotype and stage; Min, minimum average values for each variable among the 12 genotypes; Max, maximum average values for each variable among the 12 genotypes; CE/OR, ratio of cell expansion and orange-red stages (min and max) for each genotype; NA, not available; NS, non-significant (*P* > 0.05).

*0.01 <*P* <0.05; **0.001 <*P* <0.01; ***P < 0.001.

The volume of the 424 protein spots was compared among the 12 genotypes. The genes corresponding to most of these spots are identified (Xu *et al.*, 2013*a*; Supplementary Table S6 at *JXB* online). They include 133 protein spots related to primary metabolism. Several multispot proteins (one gene corresponding to several spots) were detected, such as acid invertase (seven spots), phosphoglucomutase (five spots), and enolase (five spots). These multispots may be caused by post-transcriptional and post-translational modifications or by allelic variations (Xu *et al.*, 2013*a*). A large range of variability was observed among genotypes and between stages for all the protein spot amounts (Supplementary Table S6). As for metabolites and enzymes, the main differences were observed between stages (84% significantly variable spots; Supplementary Table S6), with 46% in a lower amount and 38% in a higher amount at OR. When the data were analysed stage by stage, 256/424 spot amounts were significantly different among genotypes at CE and 274/424 at OR.

**Fig. 3.**
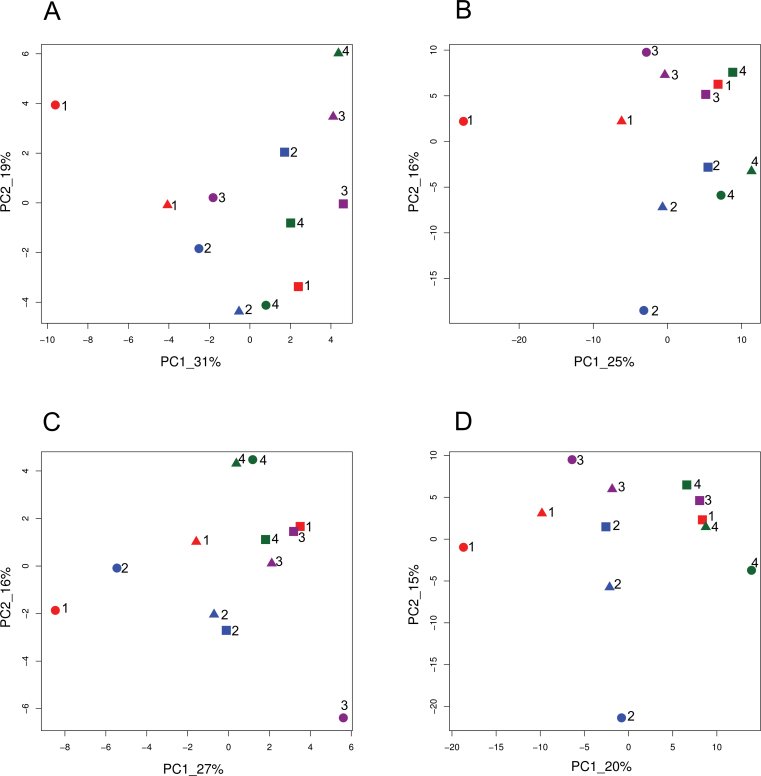
First plans of the principal component analysis showing the variation of 12 genotypes based on (A) metabolite contents, enzyme activities, and phenotypic traits at the cell expansion stage; (B) proteins at cell expansion; (C) metabolite contents, enzyme activities, and phenotypic traits at the orange-red stage, and (D) proteins at the orange-red stage. Values along the axes indicate the percentage of total variation accounted for by each component. Genotypes are indicated with different symbols, *S. lycopersicum* squares, *S. lycopersicum* var. *cerasiforme* circles, and F_1_ triangles. Levovil×Cervil (1), Stupicke Polni Rane×Criollo (2), LA0147×Plovdiv 24A (3), Ferum×LA1420 (4). (This figure is available in colour at *JXB* online.)

The variation among the 12 accessions at the different levels was illustrated by PCA. When the phenotypic traits, metabolite, and enzyme profiles were analysed at both stages, two main groups corresponding to each stage of development were detected (Supplementary Fig. S3A at *JXB* online). Similar results were obtained for the protein spot volumes (Supplementary Fig. S3B). PCAs were thus computed stage by stage (Fig. 3). In every case, Cervil (the accession with the smallest fruits) was separated from the other genotypes, and the large fruited accessions (Levovil, LA0147, and Ferum) were grouped together. Hybrids were usually located in between their parental lines.

### Inheritance of traits is predominantly additive

**Fig. 4.**
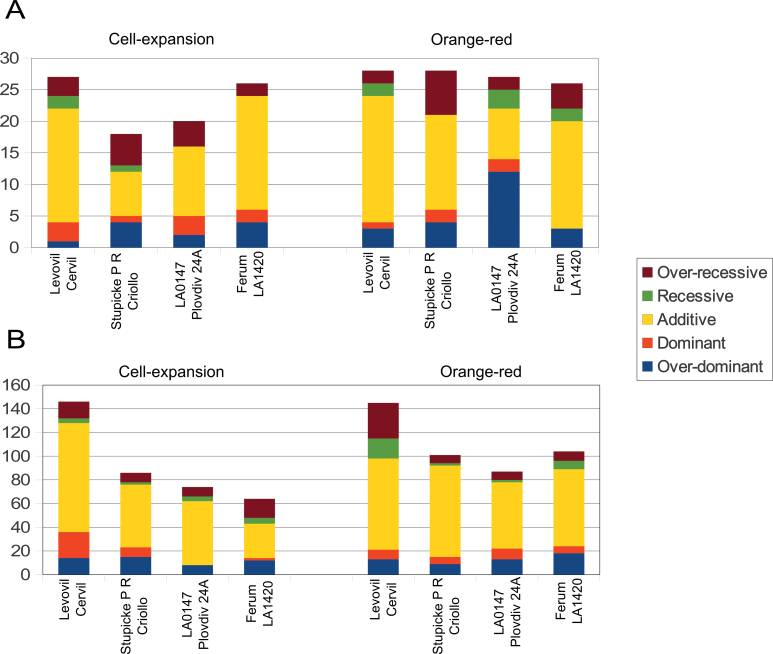
Inheritance mode of the two groups of traits in the four crosses: (A) metabolite contents, enzyme activities, and phenotypic traits; (B) protein spot volumes. From top to bottom: over-recessive, recessive, additive, dominant, and overdominant. Left panels, cell expansion stage; right panels, orange-red stage. (This figure is available in colour at *JXB* online.)

The four F_1_ hybrids were derived from crosses among the eight lines and corresponded to different distances among parental lines. The mode of inheritance of the traits that were significantly different for each cross is assessed separately (Fig. 4; Supplementary Table S7 at *JXB* online).

**Fig. 5.**
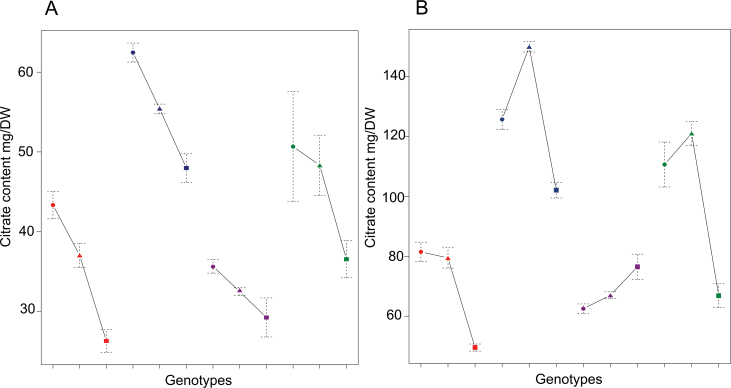
Inheritance of citrate content in tomato fruit at (A) the cell expansion stage and (B) the orange-red stage. Genotypes are indicated with different symbols, *S. lycopersicum* squares, *S. lycopersicum* var. *cerasiforme* circle, and F_1_ triangles. From left to right: Levovil×Cervil, Stupicke Polni Rane×Criollo, LA0147×Plovdiv 24A, and Ferum×LA1420. (This figure is available in colour at *JXB* online.)

The phenotypic traits fruit diameter and fruit weight were additive in the four crosses at each stage. The DMC was additive, over-recessive, or not significant according to the stage and cross (Fig. 1). Most of the metabolic contents were significantly variable at both stages. A large number of additive traits was found in the cross between the most distant lines (Levovil×Cervil) (Fig. 4A). Traits could exhibit different inheritance modes at the two stages for the same cross or in different crosses, as illustrated for the citrate content in Fig. 5.

Most of the enzyme activities were not significantly variable within one cross, so the inheritance mode of only a few enzyme activities was assessed. At CE, Ferum×LA1420 was the most variable cross, with four significantly variable enzymes, while for the other crosses, only one or no enzyme was variable. At OR, the predominant inheritance mode was additivity for Levovil×Cervil and Stupicke Polni Rane×Criollo, but not for the two other crosses.

The number of significantly variable protein spots varied among crosses at CE in relation to the genetic distance between the parental lines (Fig. 4B). As for the metabolites, proteins showed different inheritance patterns at the two stages in the same cross or in different crosses. On average, 40% of the variable traits showed a non-additive mode of inheritance without bias against recessivity or dominance.

### Dissection of relationships among traits

**Fig. 6.**
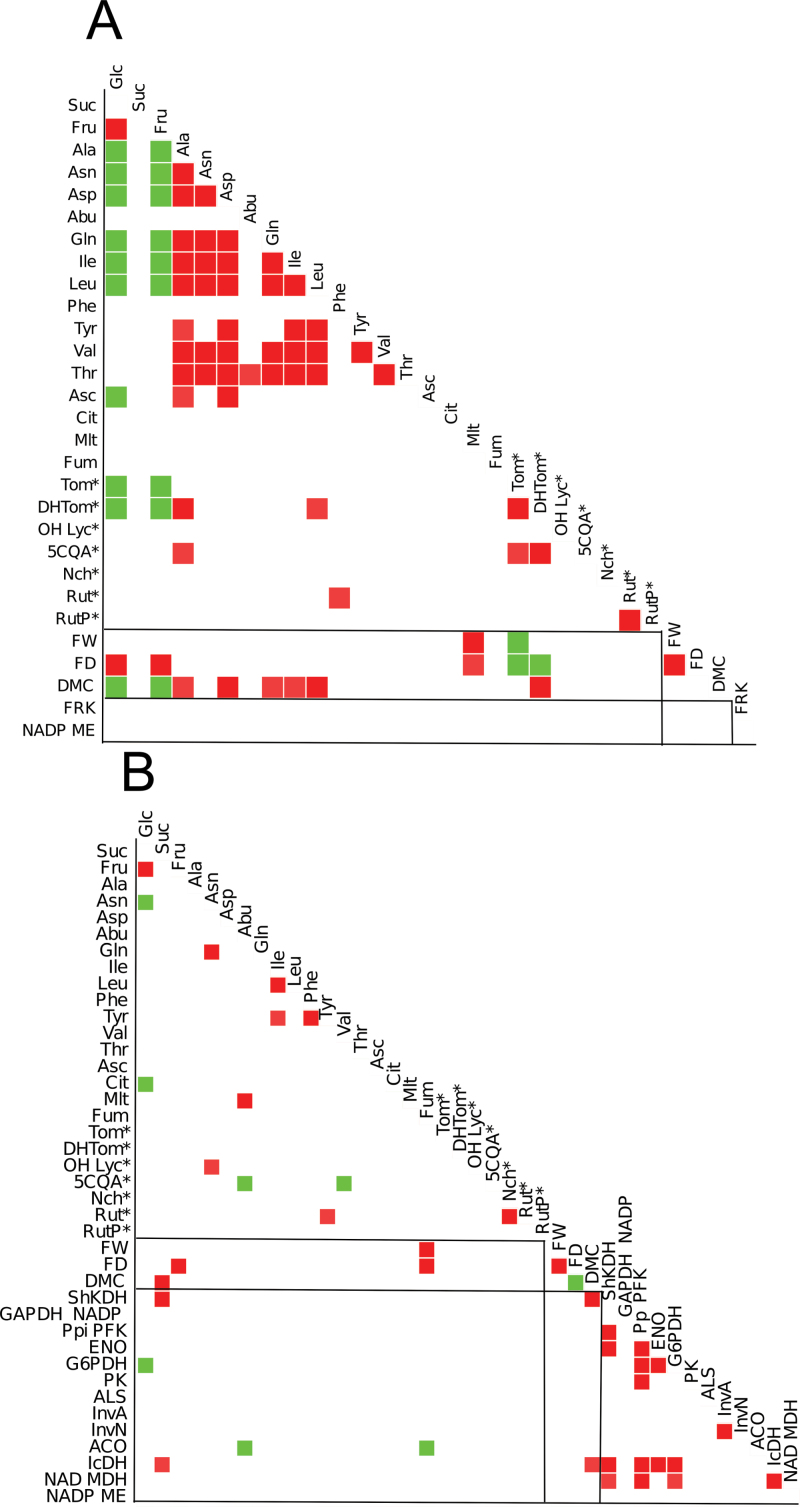
Correlations among significantly variable phenotypic and metabolic traits, and enzyme activities at (A) the cell expansion stage and (B) the orange-red stage. Only correlations where |*r*|> 0.7 (*P*-value<0.01) are shown. Dark grey (red) indicates positive correlations; light grey (green) negative correlations. (This figure is available in colour at *JXB* online.)

Relationships among traits were only assessed among the traits which were significantly different between genotypes at each stage (Supplementary Tables S8, S9, S10 at *JXB* online). The significant correlations (summarized in Supplementary Table S8) were more frequent than expected by chance, with an excess of positive correlations. Correlations among metabolites, phenotypic traits, and enzymes activities are illustrated in Fig. 6. At CE, sugars (glucose and fructose) were highly correlated with each other, and negatively with most amino acids, tomatine, and DMC. Amino acid contents were highly correlated with each other (Fig. 6A). Very few correlations were detected between metabolites and phenotypic traits at OR (Fig. 6B). For enzyme activities, correlations were significant between the glycolysis and TCA cycle enzymes at OR (Fig. 6B). Very few correlations were significant between enzyme activities and metabolite contents. Protein spot volumes were more frequently correlated with metabolite contents at CE and with enzyme activities at OR, where a large number of positive correlations with spots annotated as primary metabolism and stress response was detected. Correlations are summarized in Supplementary Table S8 at *JXB* online and provided in Supplementary Tables S9 and S10. Correlations between protein spots corresponding to enzymes and their enzyme activities were analysed at OR. In total, 28 spots corresponding to eight enzymes were analysed (Supplementary Table S11). Significant correlations, ranging from *r*=0.877 to 0.715, were detected between enolase activity and the four spots annotated as two enolase genes (Solyc09g009020 and Solyc10g085550), aldolase activity, and one aldolase gene (Solyc09g009260), and acid invertase and two spots corresponding to the Solyc03g083910 acid invertase gene. For these enzymes, even when correlations were not significant (*P* <0.01), they were often positive, with a *P*-value <0.05 (Supplementary Fig. S4). The other enzymes analysed, pyruvate kinase, glyceraldehyde-3-phosphate dehydrogenase (NAD), isocitrate dehydrogenase, malic enzyme (NADP), and malate dehydrogenase, were not significantly correlated with their corresponding spot volumes.

Table 3 lists the protein spots whose volume was strongly correlated with fruit weight or DMC. The numbers of spots were equivalent at both stages, with seven of the 15 spots related to stress response (heat shock proteins, NifU like protein, and chaperonin). A larger proportion (13/32) of correlations was detected between DMC and spots related to primary metabolism (fructokinase, malate dehydrogenase, acid invertase, and enolase). Most of the correlations with DMC were positive, in contrast to those with fruit weight.

**Table 3.** Spots highly correlated with fruit weight (a) and dry matter content (b) in eight tomato accessions and four F_1_ hybrids Pearson correlations are indicated with spot volumes assessed at the cell expression (rCE) or orange-red (rOR) stage. Range of variation of the spots at the same stage (min and max) in the 12 genotypes. Only highly significantly correlated spots (*P*-value <0.001 and |*r*| >0.78) are indicated.

	Spot	Gene	Annotation	Process			
a. Spots correlated with fruit weight							
Cell expansion					rCE	CE.min	CE.max
	JX031	Solyc08g076970.2.1	Acetylornithine deacetylase	Macromolecule	–0.82	0.31	0.55
	JX074	Solyc08g082820.2.1	Heat shock protein	Stress response	–0.88	0.75	1.21
	JX141	Solyc03g082920.2.1	Heat shock protein	Stress response	–0.83	0.46	1.00
	JX152	Solyc06g075010.2.1	chaperonin	Macromolecule	–0.81	0.57	0.87
	JX289	Solyc01g079220.2.1	NifU like protein	Stress response	0.89	4.82	14.57
	JX390	Solyc05g053470.2.1	chaperonin	Macromolecule	–0.81	1.56	2.16
Orange-red					rOR	OR.min	OR.max
	JX051	Solyc03g083910.2.1	Acid beta-fructofuranosidase	Primary metabolism	–0.84	0.24	0.71
	JX059	Solyc06g083790.2.1	Succinyl-CoA ligase	Primary metabolism	0.84	1.66	4.11
	JX135	Solyc01g057000.2.1	Universal stress protein family protein	Stress response	–0.88	2.00	3.85
	JX149	Solyc08g082820.2.1	Heat shock protein	Stress response	–0.83	0.26	0.56
	JX157	Solyc08g082430.2.1	Nucleoside diphosphate kinase	Primary metabolism	–0.80	0.58	1.18
	JX164	Solyc01g106430.2.1	Inorganic pyrophosphatase family protein	Primary metabolism	–0.80	1.12	1.69
	JX188	Solyc05g008460.2.1	ATP synthase subunit beta	Regulation	–0.87	2.43	3.41
	JX317	Solyc01g104170.2.1	Ankyrin repeat domain-containing protein 2	Regulation	0.95	0.41	0.76
	JX367	Solyc08g076220.2.1	Phosphoribulokinase/uridine kinase	Primary metabolism	0.84	0.59	1.15
b. Spots correlated with dry matter content							
Cell expansion					rCE	CE.min	CE.max
	JX012	Solyc06g005940.2.1	Protein disulphide isomerase	Macromolecule	0.86	0.25	0.53
	JX028	Solyc02g080420.2.1	RNA binding protein 45	Regulation	–0.83	0.49	0.96
	JX040	Solyc09g089580.2.1	1-Aminocyclopropane-1-carboxylate oxidase	Maturation	0.83	0.35	1.32
	JX056	Solyc01g099190.2.1	Lipoxygenase	Primary metabolism	0.87	0.31	0.73
	JX120	Solyc06g005940.2.1	Protein disulphide isomerase	Macromolecule	0.83	0.95	1.66
	JX137	Solyc06g005940.2.1	Protein disulphide isomerase	Macromolecule	0.92	0.18	0.40
	JX154	Solyc03g115990.1.1	Malate dehydrogenase	Primary metabolism	0.84	1.41	2.36
	JX218	Solyc06g073190.2.1	Fructokinase-like	Primary metabolism	0.81	3.46	5.84
	JX219	Solyc02g082800.2.1	Ubiquilin-1	Macromolecule	0.79	0.76	1.12
	JX311	Solyc12g044740.1.1	Ubiquitin carboxyl-terminal hydrolase	Primary metabolism	0.81	0.40	1.02
	JX325	Solyc01g011000.2.1	Eukaryotic translation initiation factor 5A	Translation	0.86	6.29	10.83
	JX359	Solyc04g011400.2.1	UDP-glucose 4-epimerase	Cell wall	0.79	1.72	2.44
	JX363	Solyc02g091490.2.1	Fructokinase 3	Primary metabolism	–0.84	0.44	1.13
	JX377	Solyc09g090980.2.1	Major allergen Mal d 1	Stress response	0.82	3.37	6.87
	JX395	Solyc02g086730.1.1	50S ribosomal protein L12-C	Translation	0.81	2.70	4.32
Orange-red stage					rOR	OR.min	OR.max
	JX032	Solyc01g094200.2.1	NAD-dependent malic enzyme 2	Primary metabolism	0.89	0.62	0.97
	JX035	Solyc02g078540.2.1	Unknown protein	Unknown	0.91	0.41	1.43
	JX051	Solyc03g083910.2.1	Acid beta-fructofuranosidase	Primary metabolism	0.81	0.24	0.71
	JX085	Solyc09g015000.2.1	class I heat shock protein	Stress response	0.89	0.25	4.38
	JX091	Solyc05g050120.2.1	Malic enzyme	Primary metabolism	0.93	0.10	0.70
	JX100	Solyc10g083650.1.1	Peroxiredoxin ahpC/TSA family	Oxidation–reduction	0.85	2.00	4.16
	JX103	Solyc08g075210.1.1	Acyltransferase-like protein	Regulation	0.83	0.21	0.55
	JX112	Solyc06g009020.2.1	Glutathione S-transferase	Stress response	0.79	0.95	1.89
	JX127	Solyc10g084050.1.1	26S protease regulatory subunit 6B homolog	Macromolecule	0.81	0.85	1.29
	JX139	Solyc05g056230.2.1	Calreticulin 2 calcium-binding protein	Macromolecule	0.79	0.29	0.70
	JX160	Solyc12g010040.1.1	Leucyl aminopeptidase	Macromolecule	0.79	0.32	0.68
	JX214	Solyc09g009020.2.1	Enolase	Primary metabolism	0.82	2.24	3.83
	JX223	Solyc01g106320.2.1	Octicosapeptide/Phox/Bem1p domain protein	Unknown	–0.91	0.19	0.49
	JX239	Solyc03g083910.2.1	Acid beta-fructofuranosidase	Primary metabolism	0.87	1.32	10.71
	JX262	Solyc06g068860.2.1	Alpha-mannosidase	Primary metabolism	–0.84	0.33	0.95
	JX311	Solyc12g044740.1.1	Ubiquitin carboxyl-terminal hydrolase	Primary metabolism	0.85	0.37	0.88
	JX320	Solyc12g009060.1.1	Charged multivesicular body protein 2a	Localization	0.82	0.33	0.65

### Reconstruction of networks integrating metabolic and protein profiles

Due to the large number of traits and correlations, sPLS regression was used for integrating protein expression data and metabolites, enzymes, and phenotypes. sPLS regression is a bidirectional multivariate regression method that allows separate modelling of covariance between two data sets. The main advantage of sparse methods over non-sparse methods is that it sets the contribution of noise variables to zero to improve the prediction or classification performance (Filzmoser *et al.*, 2012). sPLS networks relating protein spot volumes to phenotypes, metabolites, and enzymes were constructed at each stage. The three levels (phenotypes, metabolites, and enzymes) considering they represent a global metabolic-related level, were grouped as being related to the proteome level.

At CE, a network was constructed between metabolites, phenotypic traits, and enzyme activities (variable among genotypes) on one hand, and 77 variable protein spots related to primary and secondary metabolism and vitamin synthesis on the other hand. The network reconstructed connected eight traits and 26 proteins by >50 edges (Supplementary Fig. S5, Table S12 at *JXB* online), among which two fructokinase spots were connected to glucose, fructose, and DMC.

A network connecting variable metabolites, phenotypic traits, and enzyme activities with 87 protein spots (related to primary and secondary metabolism and vitamin synthesis) was also constructed for the OR stage. Two main networks were obtained, with more connections than for the CE stage (Supplementary Fig, S6, Table S13 at *JXB* online). Sucrose and DMC played a pivotal role. They were linked to 11 and 17 proteins, including spots corresponding to acid invertase, enolase, malate dehydrogenase, and malic enzyme

**Fig. 7.**
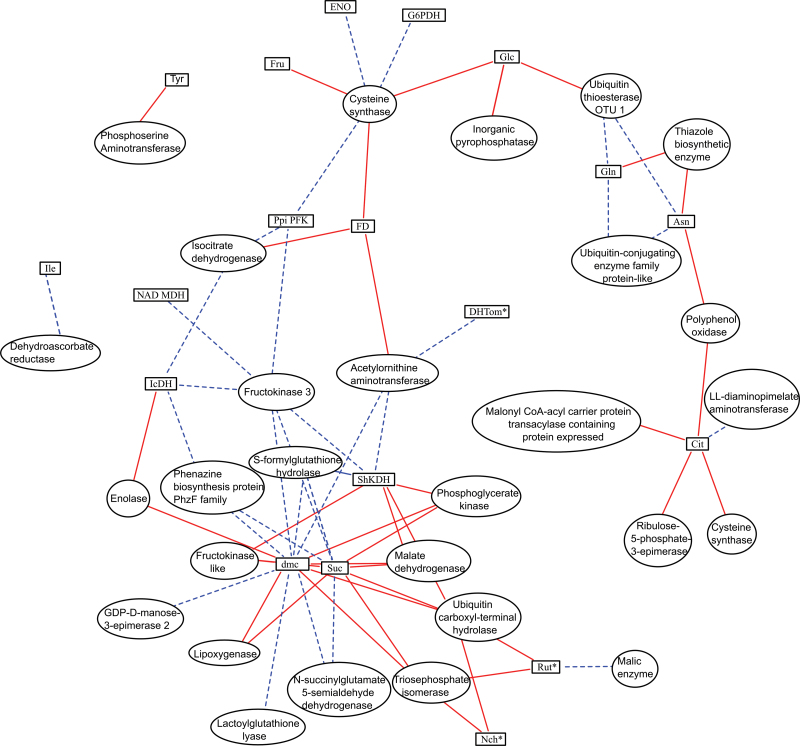
Network reconstruction based on sPLS between protein spot volumes at the cell expansion stage (circular nodes) and metabolite contents, phenotypes, and enzyme activities at the orange-red stage (square nodes). Positive and negative relations are shown bu solid and dotted lines, respectively. Annotation of spots is detailed in Supplementary Table S14 at *JXB* online. (This figure is available in colour at *JXB* online.)

As the variation of proteins expressed during CE may influence the metabolome and activome at a later stage, the connections between protein variations at CE and fruit composition and enzyme activities at OR were analysed (Fig. 7; Supplementary Table S14 at *JXB* online). A relationship was detected between sucrose content at OR and the volumes of two spots corresponding to fructokinase at CE. In addition, those spots were also related to the fructose content at CE (Supplementary Fig. S5). An isocitrate dehydrogenase spot was related to isocitrate activity at OR, and phosphoglycerate kinase from the Calvin cycle to shikimate dehydrogenase activity, an enzyme downstream of the erythrose-4P produced in that cycle. The amounts of cysteine sysnthase and fructokinase 3 proteins had a pivotal role, each being connected to several traits.

Networks were also constructed between phenotypes, metabolites, and enzyme activities and the proteins corresponding to other functions (data not shown). The most interesting relationship involved a chaperonin (JX383) that played an important role at CE, as it was related to six enzymes activities, and to glucose and fructose content at OR.

## Discussion

The variation of tomato fruit composition has been widely studied, due to its role in sensory and nutritional value. However, until now the variation of metabolic compounds has been studied in tomato either during fruit development or according to environmental perturbations, mainly in one accession or in lines resulting from the introgression of genome fragments from a unique wild species (Schauer *et al.*, 2006; Steinhauser *et al.*, 2010). The results are subsequently supposed to represent the variation of the species. In the present study, the aim was to analyse the actual variation of the species, by comparing eight accessions selected to represent a large part of the phenotypic and molecular diversity of *S. lycopersicum* (Ranc *et al.*, 2008). The variation, the inheritance, and the relationships among metabolic, enzymatic, and proteomic traits assessed at two developmental stages were described.

### A large range of genetic variation is detected at all levels

A large range of variation was observed for most of the phenotypic and metabolic traits at least at one stage. Usually, the ratio of maximum to minimum values among genotypes varied in the range of 2–3, showing that there was wide variation present in the species. The secondary metabolites showed a higher range of variation, some of them being present in one line and almost absent in another. This may be due to the inclusion in the study of *S. l.* var *cerasiforme* accessions which are not fully domesticated, as domestication caused great alterations in those compounds (reviewed by Meyer *et al.*, 2012). Among enzyme activities, significant differences were detected between stages, and the genetic variation was less significant at CE. Steinhauser *et al.* (2011) observed the same tendency, with enzyme activities having a lower heritability than metabolites, suggesting that metabolites have a tight regulation, while enzyme activities can be compensated by coordinated changes in other enzymes.

To date, proteome variation in tomato fruit and inheritance of protein amount were poorly documented (reviewed by Faurobert *et al.*, 2013). Faurobert *et al.* (2007) described the proteome variation of tomato pericarp in one line during fruit development. They could identify the function of 90 spots. Previously 424 protein spots that were variable among stages or genotypes were studied (Xu *et al.*, 2013*a*). Thanks to the release of the tomato genome sequence, the function of almost every spot was identified and 307 unique proteins corresponding to 424 spots were detected. Most of the spots which were variable at both stages showed the same tendency (an increase or decrease during fruit development) in all the genotypes. Nevertheless, 57% of the spots revealed significant genotype by stage interactions, indicating that the trend observed in one genotype at a given physiological level (stage) may change in another genotype.

The observed variation may be related to the genetic distance among accessions. In the PCA, all the large-fruited lines closely related at the molecular level were grouped together, while the small cherry tomatoes, genetically more diverse, were more spread out. In addition, Cervil, the line most distant from all the others at the molecular level, presented a very specific profile for every trait, leading to most of the extreme values (lowest fruit weight, and highest dry matter, sugar, and acid contents). It was also specific in terms of secondary metabolites, with a high content of chlorogenic acid, dehydrotomatin, and rutin. The large variation detected and the differences between genotypes during fruit development showed the important effect of genetic diversity in fruit composition and enhances the value of the presented data set.

### Diversity in the modes of inheritance among crosses and traits

Hybrids are widely used in modern agriculture, either for heterosis (the advantage of a hybrid compared with both parents) or for the combination of dominant traits. Agronomical traits often show heterosis in the F_1_ generation when distant cultivated accessions or cultivated and wild species are crossed (Springer and Stupar, 2007; Li *et al.*, 2008). The molecular origin of heterosis has been studied for years and is usually related to a combination of dominance or overdominance effects and to epistatic interactions (Stuber, 2010).

In tomato, fewer traits than in maize, a highly heterotic crop, show a systematic heterosis trend (Lippman and Zamir, 2007). Steinhauser *et al.* (2011) studied the enzyme activities in introgression lines derived from the wild species *S. pennellii* and found an approximately equivalent ratio of QTLs showing additive, recessive, and dominant modes of inheritance, with only 5% showing overdominance. In the present study, the number of traits which were significantly variable within each cross (one hybrid and its two parents) differed from one cross to the other and was related to the genetic distance at the proteomic level. Regarding phenotypic traits, in accordance with the absence of heterosis, fruit weight and diameter were additive in the four crosses. Around 60% of the other traits showed an additive inheritance, with a number of traits exhibiting an overdominant or over-recessive mode of inheritance, but no specific trend towards either one of them. The higher rate of additivity in this study compared with previous studies involving *S. pennellii* introgression lines (Schauer *et al.*, 2008; Steinhauser *et al.*, 2011) may result from the smaller distance between the parental lines, which are all from the same species.

The inheritance mode in one cross was not systematically the same in another cross and appeared relatively independent from one stage to the other, as a consequence of the complex genetic control of the traits studied. Enzyme activities, for example, are suggested to be controlled by a network of trans-acting genes (Steinhauser *et al.*, 2011); thus, dealing with different genetic backgrounds that carry different combinations of haplotypes may lead to different inheritance modes.

### A systems approach revealed complex connectivity among the different levels analysed

The genetic variation was dissected at several levels, from phenotype to metabolite and proteome profiles, in eight unrelated tomato accessions and four F_1_ hybrids at two developmental stages. In such an experimental design, a significant correlation may reveal the effect of a polymorphic gene acting on two related traits, but also fortuitous association, as a correlation between two traits may not be due to a causal relationship but to linkage disequilibrium between genes controlling the variation of both traits. Nevertheless, Osorio *et al.* (2011) in a similar approach described co-varying genes or proteins as ‘guilty by association’, as the closer the functions implicated the more meaningful were the relationships. In addition, the two stages studied in the present study correspond to very distinct physiological processes (Gillaspy *et al.*, 1993; Giovannoni, 2004), increasing the complexity but also the impact of the study.

At every expression level, more significant correlations than expected by chance were detected. The bias towards positive correlations among enzyme activities and metabolites suggested a coordinated regulation of these traits. Some of the detected relationships were already established in previous studies, such as for instance the coordinated variation between several amino acids and sugars (fructose and glucose) at CE (Prudent *et al.*, 2010).

Different studies have tried to uncover the relationships between different levels of traits (phenotypes, metabolites, enzymes, and transcripts) in tomato (Carrari *et al.*, 2006; Schauer *et al.*, 2006). As the enzyme activities assessed correspond to *V*_max_, and thus mainly reflect the corresponding protein amount, one might hypothesize that proteins and enzyme activities should be correlated. Correlations were only found between three out of eight enzymes and the protein spot amount corresponding to the same function at OR, all of them showing a positive correlation, as expected from a causal relationship. This lack of relationship between enzyme activities and their protein amounts is consistent with the results obtained when comparing enzyme activities and their corresponding gene expression (Gibon *et al.*, 2006; Morcuende *et al.*, 2007; Steinhauser *et al.*, 2010). This may be due to the fact that enzyme activities result from a combination of several proteins (subunits) or that most of the primary metabolism enzymes belong to multigene families. In addition, protein spots may also be the product of complex post-translational modifications, where only one of the forms will be the functional one (Faurobert *et al.*, 2007).

One problem when dealing with omic data is that the number of traits is much larger than the number of samples. Sparse methods were developed for dealing with high-dimensional data. Such a method sets the contribution of noise variables to zero and thus improves the prediction of correlations or classification performance (Filzmoser *et al.*, 2012). The networks reconstructed with sPLS methods showed complex patterns of connectivity, relating several nodes together and different pathways or metabolisms. In each network, a few hubs could be identified relating many different compounds or proteins.

At CE, several correlations with DMC and metabolite contents involved two of the protein spots coding for fructokinase, an enzyme participating in sugar phosphorylation. Fructokinase plays a role in sugar import and in starch biosynthesis (Dai *et al.*, 2002). Several isoforms were detected, being correlated with the variation of sugars.

The network constructed at OR revealed the key role of invertase in sucrose breakdown, as already documented (Faurobert *et al.*, 2007). Two spots corresponding to this function were strongly related to the content in sucrose and DMC. In addition, they were also correlated with several enzyme activities and with fruit weight. Schauer *et al.* (2006) have also detected an association between phenotypic traits and metabolic compounds in tomato.

Previous studies in *Arabidopsis* suggested that the relationships between transcript modifications and enzyme activities showed greater agreement in the long term (Osuna *et al.*, 2007). The relationships between protein amounts at CE and the other traits at OR were thus analysed. Relationships between isocitrate dehydrogenase enzyme activity and the protein spots corresponding to this protein were then detected. In addition, the interconnections suggested a possible role as a regulator for cysteine synthase whose amount at CE is correlated with several enzymes, glucose, and fruit diameter at OR. This enzyme has been suggested to play a key role in highly metabolically active cells (Wang *et al.*, 2003). Those facts should be taken into account when trying to modify gene expression or protein contents in order to alter metabolite contents. According to the present findings, this approach will only work for a small subset of metabolites, so researchers should focus on proteins such as fructokinase or cysteine synthase that affect several metabolites, instead of just the enzymes that regulate the direct synthesis of a target compound.

The present analysis provided a detailed characterization of fruit metabolism, at several levels in a set of accessions representing a wide range of genetic variation, and led to interesting conclusions. First, the contents of primary and secondary metabolites are quite variable depending on the genetic background, while enzyme activities seem to be less variable, particularly at CE. In addition, significant genotype by stage interactions showed that the trends observed in one genotype at a physiological level may change in another genotype. In agreement with this, the inheritance modes varied between crosses and stages, showing the multigenic nature of the traits studied, although additivity was predominant. The network reconstruction revealed associations between different levels of expression and provided information on several key proteins that might be targets for improving metabolite contents. This study is the starting point of a broad experiment including the development of a multiallelic population derived from the eight parental lines. QTLs for fruit composition will be mapped in the population and will be related to the variations observed at various levels in the parental lines.

## Supplementary data

Supplementary data are available at *JXB* online.

Figure S1. Fruits of the eight tomato lines and four F_1_ hybrids studied.

Figure S2. Fruit fresh weight variation during fruit development in the eight lines and four F_1_ hybrids.

Figure S3. First plan of the principal component analysis showing the variation of 12 genotypes at two stages.

Figure S4. Correlations between the volumes of protein spots corresponding to acid invertase and the enzyme activity.

Figure S5. Network reconstruction based on sPLS between protein spot volumes and metabolites, phenotypes, and enzyme activities at cell expansion.

Figure S6. Network reconstruction based on sPLS between protein spot volumes and metabolites, phenotypes, and enzyme activities at the orange-red stage.

Table S1. Accession origins.

Table S2. Polymorphism rate between the eight parental lines and Heinz1706 (the tomato reference genome) detected with 139 SNPs (from Xu *et al.*, 2013*b*).

Table S3. List of the 19 primary metabolites and eight secondary metabolites analysed.

Table S4. List of the enzyme activities analysed.

Table S5. Means and standard deviations for all the traits studied.

Table S6. Annotation and analysis of variation of protein spots

Table S7. Analysis of variation cross by cross at each stage.

Table S8. Overview of the number of significant correlations within and among different levels of analysis.

Table S9. Correlations at the cell expansion stage.

Table S10. Correlations at the orange-red stage.

Table S11. Correlations between enzyme activities and the spots coding for those enzymes at the orange-red stage.

Table S12. Annotation of spots included in the cell expansion stage network (Supplementary Fig. S5)

Table S13. Annotation of spots included in the orange-red stage network (Supplementary Fig. S6)

Table S14. Annotation of spots included in the cell expansion and orange-red network (Fig. 7)

Supplementary Data
