# Supplementary material for: Deciphering genetic diversity and inheritance of tomato fruit weight and composition through a systems biology approach
Source: J Exp Bot. 2013 Oct 22;64(18):5737–52. doi: 10.1093/jxb/ert349 (PMC3871826; doi:10.1093/jxb/ert349)
Supplement: Supplementary Data [file supp_64_18_5737__index.html]

Deciphering genetic diversity and inheritance of tomato fruit weight and composition through a systems biology approach — Deciphering genetic diversity and inheritance of tomato fruit weight and composition through a systems biology approach — Supplementary Data 

# Deciphering genetic diversity and inheritance of tomato fruit weight and composition through a systems biology approach

## Supplementary Data

Data files

**Files in this Data Supplement:**

- Supplementary Data - Supplementary Data
- Supplementary Data - Supplementary Data
