# Supplementary material for: Deciphering genetic diversity and inheritance of tomato fruit weight and composition through a systems biology approach
Source: J Exp Bot. 2013 Oct 22;64(18):5737–52. doi: 10.1093/jxb/ert349 (PMC3871826; doi:10.1093/jxb/ert349)
Supplement: Supplementary Data [file supp_ert349_jexbot103796_file001.pdf]

Journal of Experimental Botany

Deciphering genetic diversity and inheritance of tomato fruit weight and composition through a systems biology approach

Laura Pascual, Jiaxin Xu, Benoît Biais, Mickaël Maucourt, Patricia Ballias, Stéphane Bernillon, Catherine Deborde, Daniel Jacob, Aurore Desgroux, Mireille Faurobert, Jean-Paul Bouchet, Yves Gibon, Annick Moing, Mathilde Causse

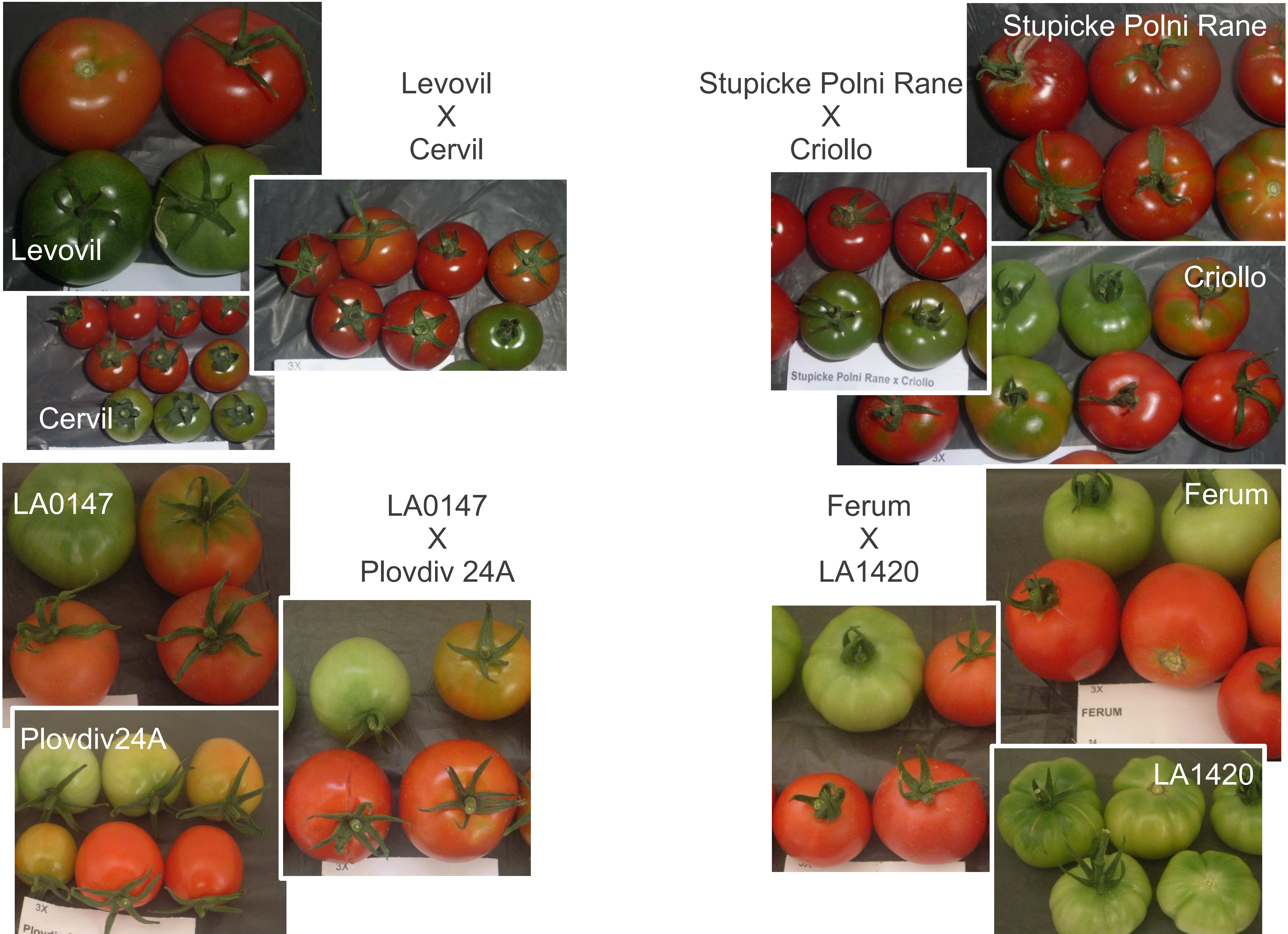

Supplementary Figure S1. Fruits of the eight tomato lines and four F1 hybrids.

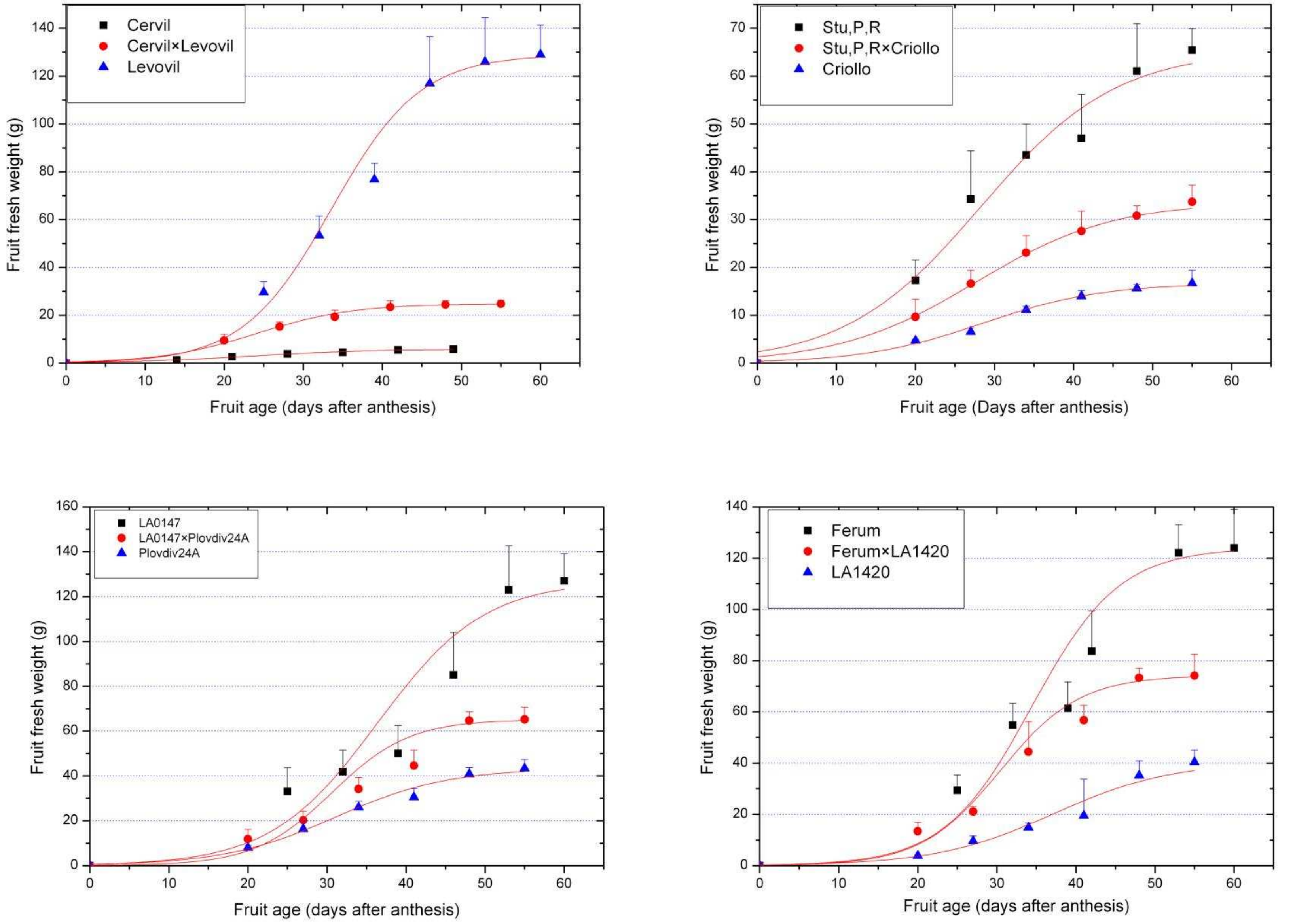

Supplementary Figure S2. Fruit fresh weight variation during fruit aging in the eight lines and four F1 hybrids.

A

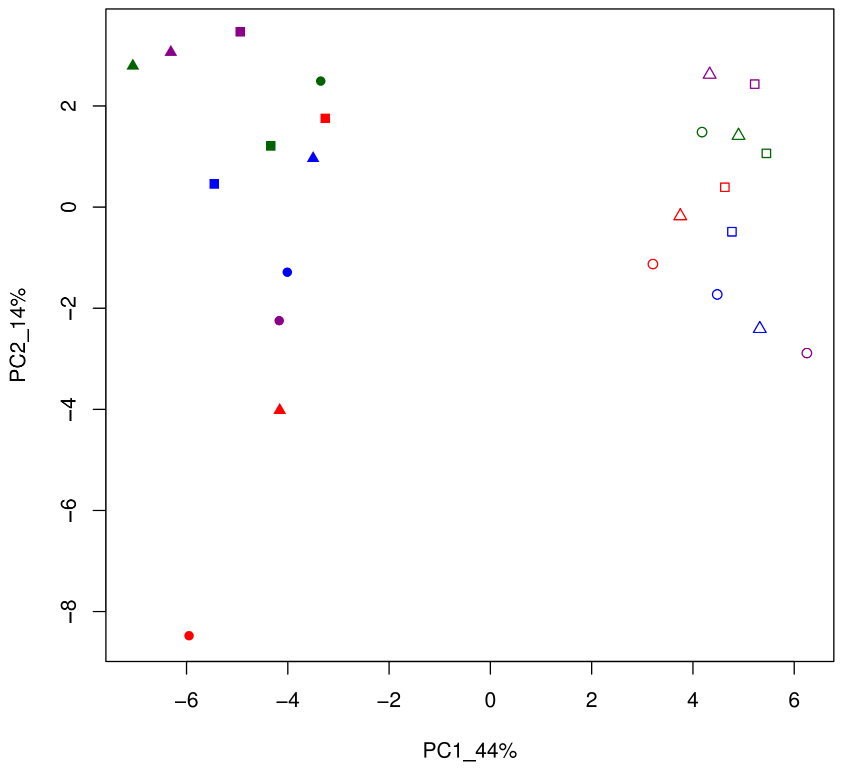

B

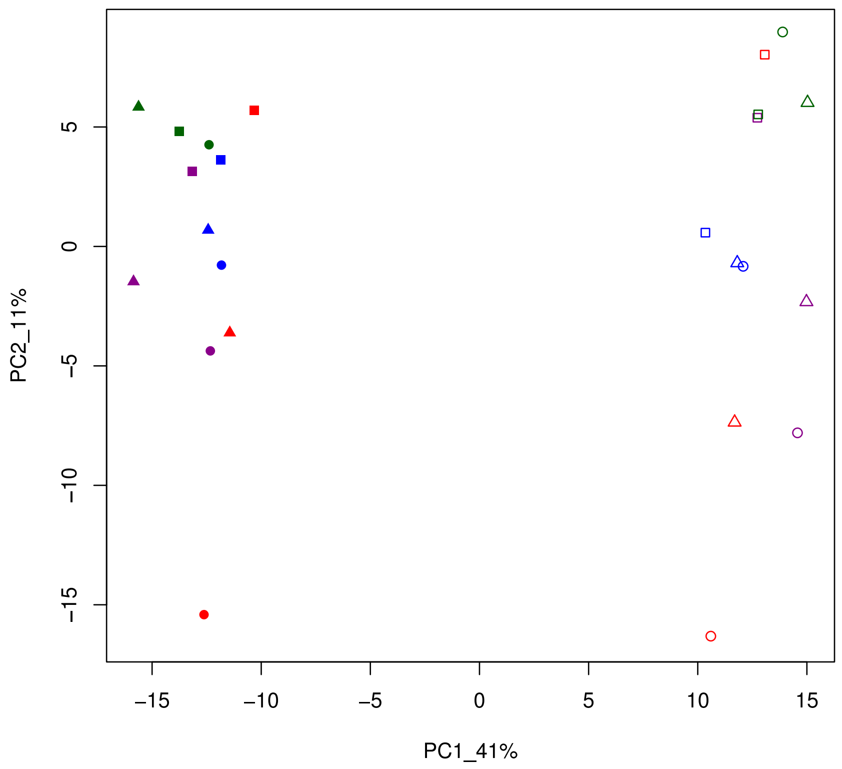

Supplementary Figure S3. First plan of the principal component analysis showing the variation of 12 genotypes at two stages based on (A) metabolites, enzymes and phenotypes; (B) proteins.
Values along the axes indicate the percentage of total variation accounted for each component.
Cell expansion samples with filled symbols and orange-red with emtpy symbols.
Genotypes are indicated with different symbols, S. lycopersicum squares, S. lycopersicum var cerasiforme circles and F1 triangles. Levovil x Cervil red, Stupicke Polni Rane x Criollo blue, LA0147 x Plovdiv 24A purple, Ferum x LA1420 green.

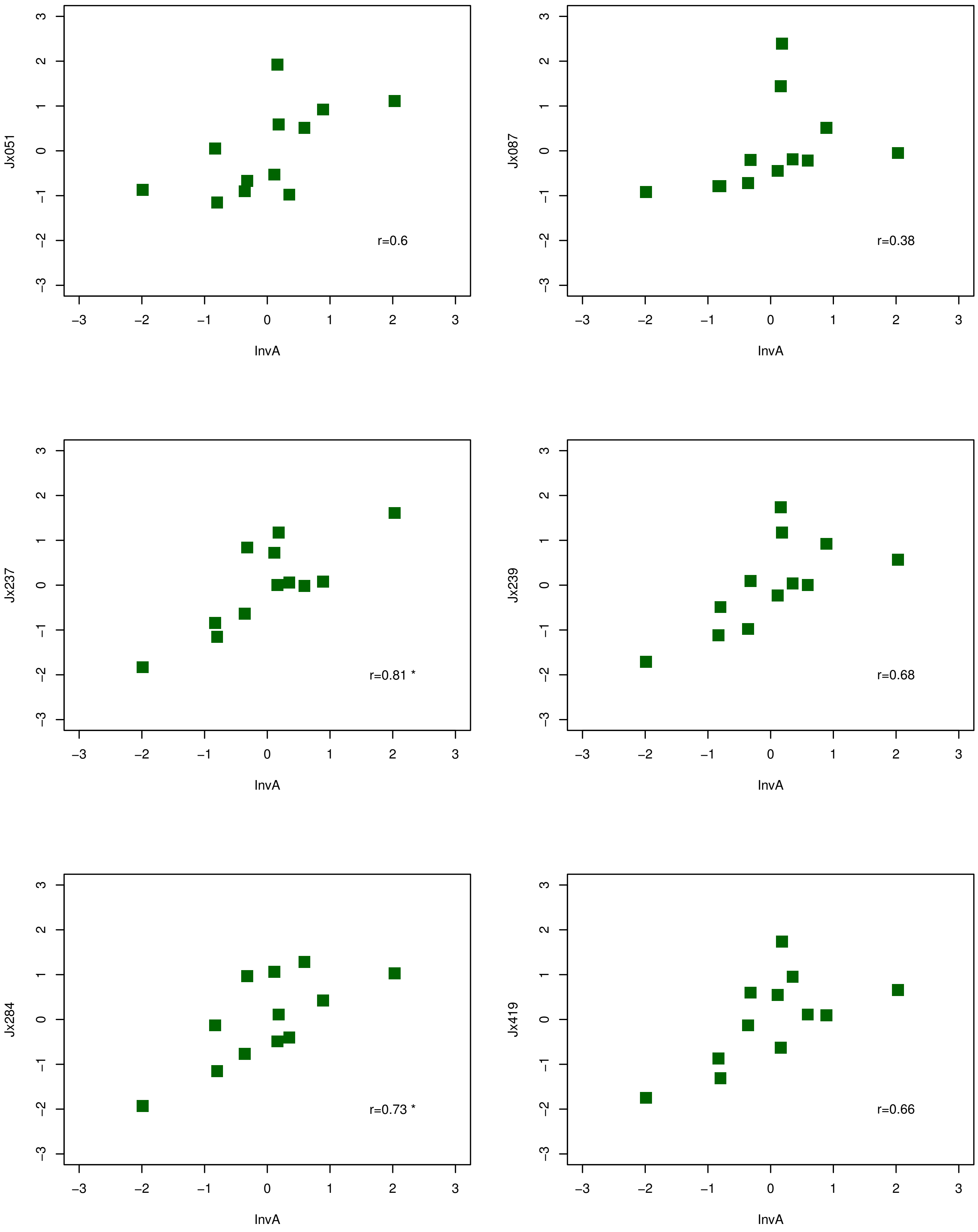

Supplementary Figure S4. Correlations between the volumes of protein spots corresponding to acid Inverstase and the enzyme activity. * Significant correlations, p-value<0.01.

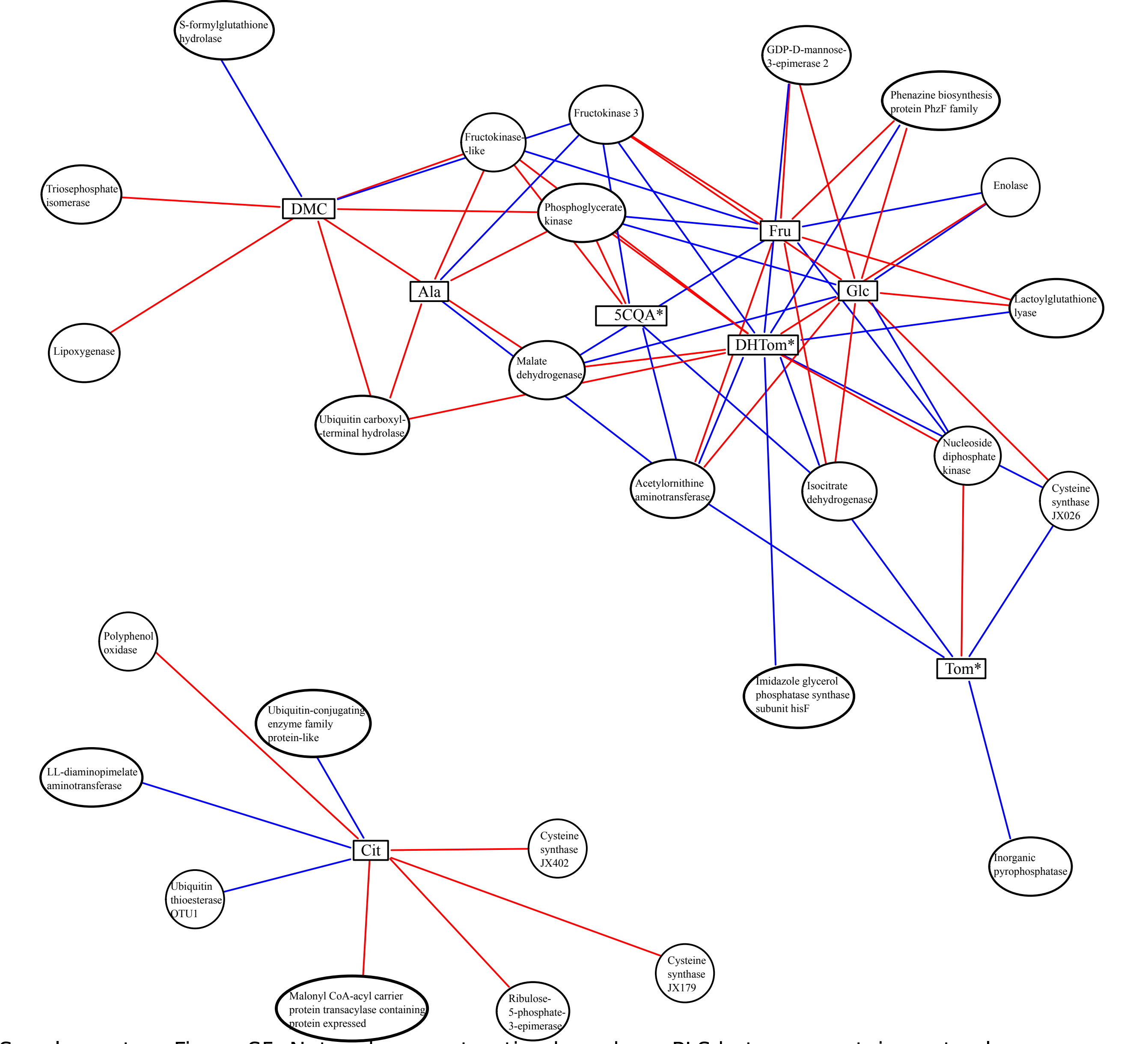

Supplementary Figure S5. Network reconstruction based on sPLS between protein spot volumes (circular nodes) and metabolites, phenotypes and enzymes (square nodes) at Cell expansion. Positive and negative relations in red and blue. Spots annotation in Supplementary Table S12. When two spots correspond to the same protein spot number is noted.

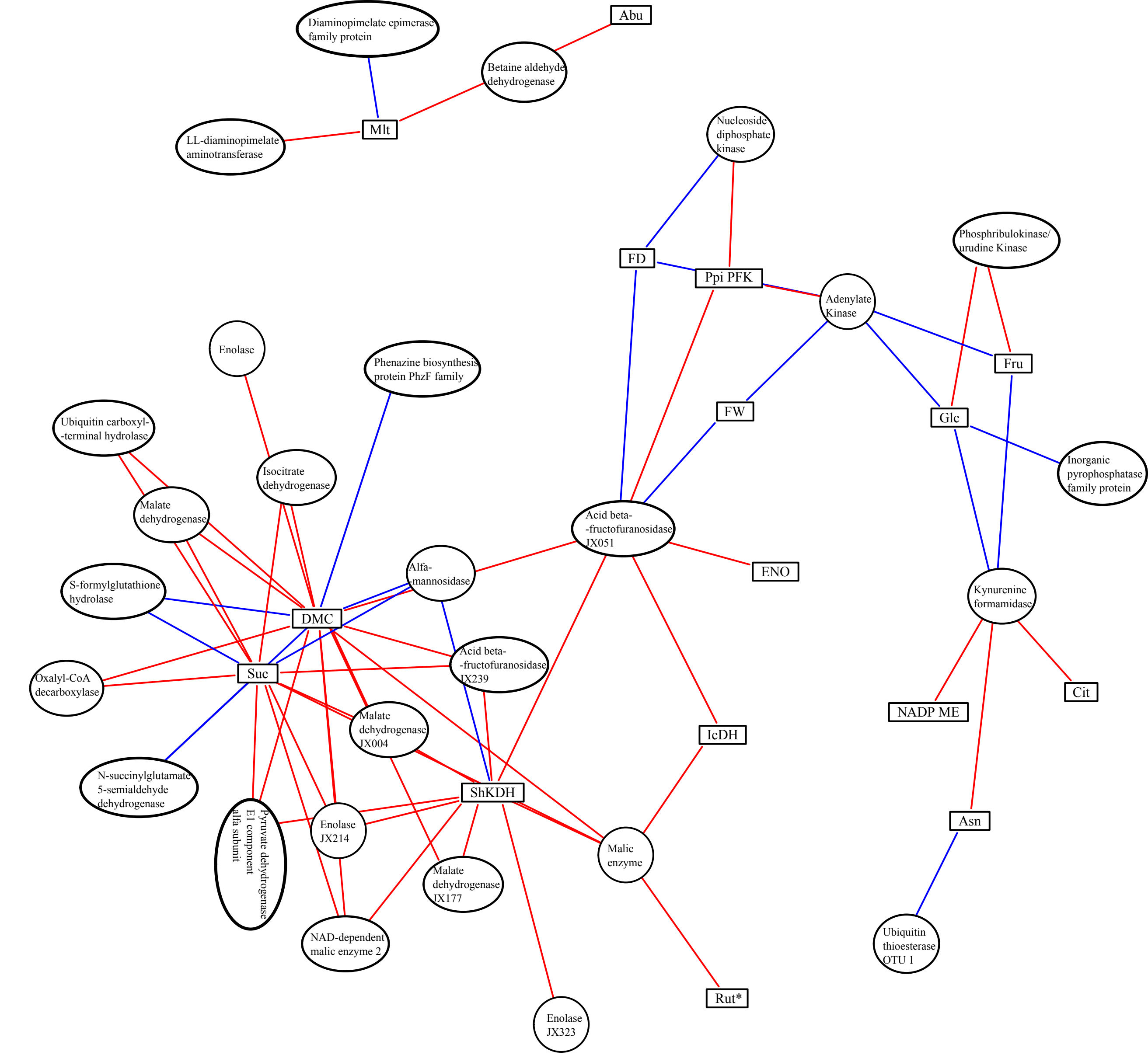

Supplementary Figure S6. Network based on sPLS between protein spot volumes (circular nodes) and metabolites, phenotypes and enzymes (square nodes) at orange-red. When two spots correspond to the same protein spot number is noted. Positive and negative relations in red and blue. Spots annotation in Supplementary Table S13.
